# Multi-scale comparison of the fine particle removal capacity of urban forests and wetlands

**DOI:** 10.1038/srep46214

**Published:** 2017-04-10

**Authors:** Zhenming Zhang, Jiakai Liu, Yanan Wu, Guoxin Yan, Lijuan Zhu, Xinxiao Yu

**Affiliations:** 1 College of Nature Conservation, Beijing Forestry University, Beijing 100083, China; 2 College of Soil and Water Conservation, Beijing Forestry University, Beijing 100083, China

## Abstract

As fine particle (FP) pollution is harmful to humans, previous studies have focused on the mechanisms of FP removal by forests. The current study aims to compare the FP removal capacities of urban forests and wetlands on the leaf, canopy, and landscape scales. Water washing and scanning electron microscopy are used to calculate particle accumulation on leaves, and models are used to estimate vegetation collection, sedimentation, and dry deposition. Results showed that, on the leaf scale, forest species are able to accumulate more FP on their leaf surface than aquatic species in wetlands. On the canopy scale, horizontal vegetation collection is the major process involved in FP removal, and the contribution of vertical sedimentation/emission can be ignored. Coniferous tree species also showed stronger FP collection ability than broadleaf species. In the landscape scale, deposition on the forest occurs to a greater extent than that on wetlands, and dry deposition is the major process of FP removal on rain-free days. In conclusion, when planning an urban green system, planting an urban forest should be the first option for FP mitigation.

Particulate matter (PM) is considered with aerodynamic diameters equal to or less than 10 μm while fine particles (FP) refer to particulates with an aerodynamic diameter range of 0.2–2.5 μm^1^; they contain highly toxic components, such as heavy metals and potential carcinogens^2^,^3^, and can be inhaled into the bronchus and deep lungs^4^, causing alveolar inflammation. In recent years, particles have led to hazy weather in many cities in China^5^,^6^,^7^. Thus, determining an effective way to reduce particle concentrations in urban areas has become a crucial endeavor. Urban green systems, including urban forests, wetlands, and green belts, have gained recognition for their contribution to air cleaning^3^, and relevant studies have mainly focused on the leaf, canopy, and landscape scales to estimate the roles of plants and urban green systems in particle removal.

Foliage surfaces are an important research hotspot because these surfaces can capture and accumulate PM. While plants are considered excellent biological filters^6^,^8^,^9^,^10^,^11^,^12^,^13^, different species show varying accumulation abilities. For example, a Polish study indicated that *Syringa meyeri* presents the strongest PM capturing ability out of all commonly planted urban trees tested^10^. In Norway, research has indicated that *Betula pendula, Pinus mugo, Pinus sylvestris*, and *Salix cinerea* show higher annual PM accumulation than other commonly cultivated species in urban areas^12^. Furthermore, research in Beijing, China, has demonstrated that while *Cephalotaxus sinensis, Euonymus japonicas, Broussonetia papyriferar, Koelreuteria paniculata*, and *Quercus variabilis* can efficiently capture particles^6^, these plants are not commonly selected afforestation species in the studied area. Varying PM accumulation abilities are related to the surface properties, hair density^12^, number of stoma^6^, growing stage^14^, presence or absence of a thin film, and number of tubules on a leaf’s surface^9^. Unfortunately, previous studies have only focused on forest species and ignored hydrophytes in wetlands, which also feature high PM accumulation potential.

On the canopy scale, tree belts have been reported to block FP and reduce its concentration in both the vertical and horizontal directions^7^,^15^,^16^,^17^,^18^. FP is collected by vegetation through impaction, interception, and sedimentation^19^. Studies in central Shanghai, China, have shown that FP concentrations decrease with increasing tree height in urban street canyons and that the rate of decrease in green streets is higher than that in tree-free streets^16^. Results of simulated experiments have shown that the FP collection speed of coniferous trees is about 7.4 μg m^−3^ s^−1^ while that of broadleaf species can reach 33.5 μg m^−3^ s^−1^ in the full-leaf stage^15^. Contrasting with these findings, research conducted next to a highway area in New York, USA, showed that the presence of a green belt between the source and the downwind area reduces the frequency and intensity of concentration spikes but that the average concentration and local FP concentration increase in relation to aerodynamics^20^. These differing results could be related to the tree belt structure, such as the leaf area index, leaf area density distribution, trunk height, tree-covered area, and tree planting pattern^21^, and the monitoring distance from the tree belt. Thus, the effect of tree belts in mitigating FP in a specific region requires further study.

Another research hotpot in FP removal involves effects on the landscape scale, which relates to dry deposition onto forests, wetlands, and other land cover types^3^,^22^,^23^,^24^,^25^,^26^,^27^. Such research has recently been especially pertinent in Beijing because of the presence of serious pollution within the city^7^,^28^,^29^,^30^,^31^. Results have determined that deposition shows temporal variation and spatial heterogeneity. For example, FP deposition onto forests is higher than that on wetlands^29^, and the deposition velocity in coniferous forests is higher than that in broadleaf deciduous forests^31^. Studies have also determined that the deposition velocity during the day is higher than that at night because higher PM concentrations and the meteorological conditions, such as relatively higher humid and wind speed, during the daytime are more favorable to dry deposition^7^,^29^.

Theoretical and monitoring research on different scales has expanded the scientific knowledge on the mechanisms and processes of FP removal by vegetation. However, practical application of scientific results to urban design and planning is now necessary to mitigate serious pollution and improve air quality, particularly in urban areas. Developing an optimized plan for a specific region that involves species selection and specific landscape construction is thus essential and necessitates conducting comparative research by field investigations or model simulations, using several alternative scenarios in the same pollutant background. The general lack of availability of models to describe FP removal has been acknowledged. Thus, against this background, the aim of the present work is to determine the most effective urban design scheme for FP removal in Beijing. Our study will (1) compare the FP leaf accumulation abilities of different forest and wetland species, (2) reveal the spatiotemporal heterogeneities in FP collection by vegetation in a green belt alongside a road on the individual scale, and (3) compare FP dry deposition onto an urban forest and wetland on the landscape scale.

## Materials and Methods

### Experiment site

**Figure 1:**
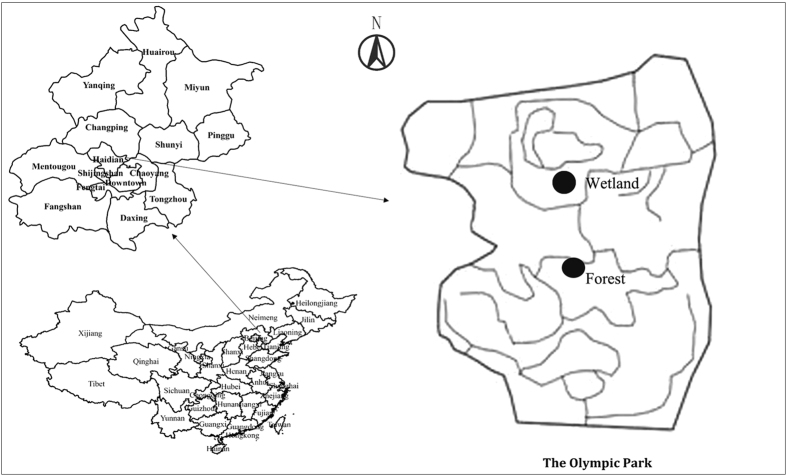
Location of experiment sites (Diagram created using ArcGIS 9.3).

As shown in Fig. 1, the experimental sites are located in an artificial wetland, an artificial forest, and a roadway in Beijing Olympic Forest Park, which is situated at the north end of the central axis of Beijing and crossed by the Beijing 5^th^ Ring Road. Both the wetland and forest sites are far away from the Ring Road to avoid the influence of traffic follow. The forest site is dominated by *Populus* × *canadensis* with some *Pinus tabulaeformis, Morus alba, Quercus variabilis, Sophora japonica*, and *Gleditsia sinensis*, all of which are commonly used species in urban planning in north China.

The average distance between two adjacent trees is about 10 m. The wetland consists of a lake with an herbaceous swamp on the shore containing *Phragmites australis, Scirpus validus, Typha orientalis, Lythrum salicaria*, and *Iris* spp. All of the species are randomly distributed throughout the forest and wetland. The roadway is located to the north of the forest site. The main wind direction is from north to south, orthogonal to the roadway; thus, the north side of the tree belt is defined as the windward side and the south side is considered the leeward side. Roadside tree species include *Populus* × *canadensis* and *Sabina chinensis.*

### Experimental design

This research aims to compare the abilities of forests and wetlands to remove FP on the leaf, canopy, and landscape scales. To determine leaf-scale removal abilities, FP accumulation was compared on the leaves of common forest tree species and aquatic plants in Beijing; to determine canopy-scale removal abilities, vegetation accumulation and sedimentation/emission within the canopy of the forest site were calculated. Finally, to determine landscape-scale FP removal, dry deposition onto the forest and wetland was estimated and compared. Vegetation collection within the wetland was ignored because of small plant sizes and the fact that nearly no difference in FP concentration was determined at the top and bottom of plants. Vegetation collection was thus compared between evergreen coniferous forest trees and deciduous broadleaf forest trees.

To evaluate leaf accumulation, six aquatic species and seven common tree species were selected. The aquatic species include *P. australis, S. validus, Iris wilsonii, T. orientalis, Iris setosa*, and *L. salicaria*; and the tree species include *M. alba, S. matsudana, Q. variabilis, Populus* × *canadensis, S. japonica, G. sinensis*, and *S. chinensis.* All leaf samples were collected twice a month from April 2014 to October 2014, and specific sampling days were decided by the following weather conditions: 7 days after precipitation of more than 15 mm and no precipitation or strong winds (wind speed >6 m/s) during the sampling week^11^,^32^. All of the sampled plants were in good condition with little or no disease or pests. During sample collection of aquatic plants, the top leaves from all directions (north-, south-, east-, and west-facing leaves), as well as tree species with a height of 6 m, which is the same height as the FM monitors installed in the forest, were used as criteria. Leaf samples measured about 40 cm^2^ in size, and 3–4 leaves were collected as one sample. Four samples were collected for each species, and all leaves were immediately sealed in a plastic bag and stored in a refrigerator (5 °C).

**Figure 2:**
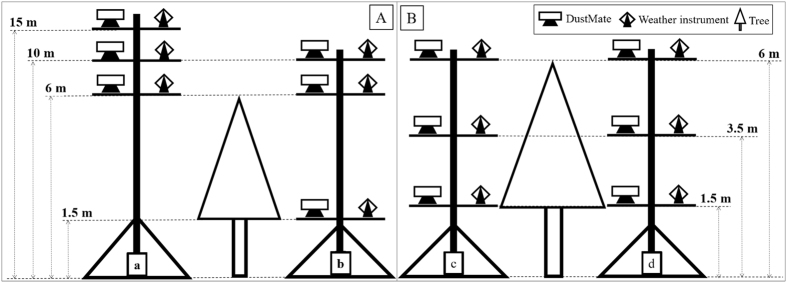
Arrangement of experiment equipment. In part (**A**) (for the landscape scale), (a) and (b) are equipment installed for dry deposition and vegetation collection in forest and wetland, respectively; in part (**B**) (for the canopy scale), (c) and (d) are leeward and windward vegetation collection, respectively (diagram created using Adobe Photoshop CS5).

During vegetation collection, as shown in Fig. 2B, FP concentration and meteorological data, including temperature, humidity, wind velocity, wind direction, and solar irradiance, were obtained from both sides of leaves at the bottom (1.5 m), middle (3.5 m), and top (6 m) of the *S. chinensis* and *Populus*×*canadensis* canopy along the roadway. Concentration data were collected by DustMate (DUSTMATE, Turnkey Co. Ltd., United Kingdom) monitors based on particle counters, and meteorological data were collected by portable weather instruments (Kestrel 4000 Pocket Weather Meter, Nielsen-Kellerman Co. Ltd., USA). Both concentration and meteorological data were automatically collected and recorded every 1 minute. We selected midsummer with lush vegetation (June–August) and early winter after defoliation but no snow or frost (October–December) as experiment seasons, and randomly chose 6 rain-free days in each season. The instruments recorded continuous data for 24 hours every sampling day (from 7:00 am of the sampling day to 7:00 am the next day), and each day was divided into daytime (from 7:00 am to 7:00 pm) and nighttime. The DustMate monitors were calibrated twice a day (7:00 am and 7:00 pm), and the sampling heads were washed by ultrasonic cleaning with deionized water three times before each sampling.

Determinations of dry deposition were conducted in the forest site and wetland (Fig. 2A). Three heights of 6 m (the average canopy height in the forest), 10 m, and 15 m were monitored in the forest, and another three heights of 1.5 m (the average plant height in the wetland), 6 m, and 10 m were monitored in the wetland site. At each height, a DustMate monitor and portable weather instrument were mounted on the trees. The sampling time and period for dry deposition are identical to those applied during vegetation collection. In addition, an iron tower with ultrasonic anemometers (Wind Master, Gill Instruments, United Kingdom) was constructed in the forest site to obtain the Monine-Obukhov length and friction velocity. A wooden plant was also constructed in the wetland center to enable installation of all of the equipment. All of the equipment was installed at the geometric center of the forest and wetland, and no endangered or protected species were involved in the field studies.

### Data analysis

#### Leaf accumulation

The water washing method was performed to calculate particle accumulation on the surfaces and wax layers of leaves. Filters were weighed before and after FP removal and then sorted using an electronic balance (BT125D, Sartorius Co., Ltd., Beijing, China) that was sensitive to 0.00001 g. The filters were then placed in a polytetrafluoroethylene balancing box under constant temperature (25 °C) and humidity (40%) for 48 h. The balancing box measured 80 cm × 80 cm × 80 cm. A balance and humidity controller (WHD48-11, ACREL Co., Ltd., Jiangsu, China) were also placed in the balancing box. A 100 cm^2^ sample from each species was clipped for washing in distilled water and chloroform to remove particles on the leaf surface and wax layer, respectively. The accumulation amount per unit area of each leaf was then used to compare accumulation capacities among species. The washing liquid was filtered using filters with a pore diameter of 2.5 μm, collected, and then passed through filters with a pore diameter of 0.2 μm. The difference before and after filtration using the 0.2 μm filters was considered equal to the amount of FP collected.

Leaf samples were scanned using a scanning electron microscope (SEM) (S-3400N II, Hitachi Japan Co., Ltd., Tokyo, Japan). Leaves were dehydrated using a dryer (DHG-9145A, Shanghai Yiheng Scientific Instrument Co., Ltd., Shanghai, China) at 60 °C for 48 h, cut into small strips (9 mm^2^), and mounted on a sample stage. The strips were photographed from different observation points on both the adaxial and abaxial surfaces at a magnification range of ×370–750 SE.

#### Vegetation collection and sedimentation

In the horizontal direction within the canopy, interception and impaction are two major FP vegetation collection processes^7^,^19^,^29^. Brownian diffusion and rebound were ignored in this research because we focused on particles ranging from 0.2 μm to 2.5 μm in size; Brownian diffusion affects very FPs (usually smaller than 0.1 μm) and rebound influences coarse particle deposition at sizes typically larger than 5 μm. Thus, the total collection velocity may be calculated as the sum of the interception and impaction velocities as follows


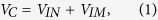


where *V*_*C*_ (cm s^−1^) is the vegetation collection velocity and *V*_*IN*_ (cm s^−1^) and *V*_*IM*_ (cm s^−1^) are the collection velocities associated with interception and impaction, respectively. These parameters are calculated as


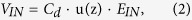



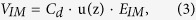


where *C*_*d*_ is the plant drag coefficient, specified as 1/6 in this research, and u(z) is the average wind velocity (m s^−1^) at height z^29^. *E*_*IN*_ and *E*_*IM*_ are the collection efficiencies of vegetation surfaces from the interception and impaction processes, respectively, and can be presented as^33^


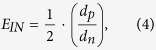



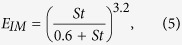


where *St* is the Stokes number, which can be presented as


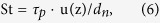


where *d*_*n*_ is the dimension of the vegetation element and given for different land cover types and seasons^34^. *τ*_*p*_ is the particle relaxation time, which can be presented as follows:


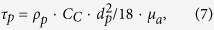


where *β* is an empirical constant (0.8 for the forest and 2.0 for the wetland with plants), *d*_*p*_ is the mean particle diameter (μm), *μ*_*a*_ is the dynamic air viscosity, *ρ*_*p*_ (μg m^−3^) is the density of the particles (and can be replaced by the particle concentration)^7^,^29^, and *C*_*C*_ is the Cunningham correlation factor, which can be calculated as follows:


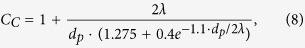


where *λ* is the mean free path of air (65 nm). The amount of vegetation collection can be calculated as follows:


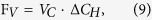


where F_*V*_ is the vegetation collection and Δ*C*_*H*_ is the difference in concentrations between the leeward and windward sides (μg m^−3^). In the vertical direction, FP movement within the canopy is affected both by gravity and vegetation properties; this movement is defined as sedimentation^19^ and can be presented as follows:


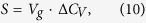



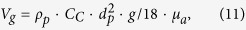


where *S* is the level of sedimentation, *V*_*g*_ (cm s^−1^) is the sedimentation velocity, and Δ*C*_*V*_ (μg m^−3^) is the concentration difference between two heights.

#### Dry deposition to the forest

Dry deposition is the process wherein particles are deposited directly from the atmosphere onto vegetation, soil, or other surfaces without the hydrometer as a medium^35^. In this work, the eddy correlation method^7^,^29^,^31^,^35^,^36^,^37^ was used to calculate dry deposition onto the forest, and deposition fluxes were calculated as follows:


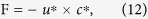


where F is the level of dry deposition (μg · s^−1^ · *m*^−2^), u* (m s^−1^) is the friction velocity, and c* (μg m^−3^) is the eddy concentration, which is calculated as follows:


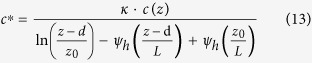


were *κ* is the Von Karman constant (0.4), *L* (cm) is the Monine-Obukhov length, *c(z*) (μg m^−3^) is the concentration at a specific height, and *ψ*_*h*_ represents the integrated stability correction function, which can be calculated as follows for stable conditions (L > 0):


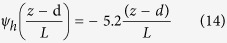


and unstable conditions (L < 0) as follows:


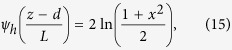



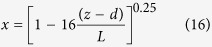


More details on these calculations can be found in Liu *et al*.^29^ and Sun *et al*.^31^.

#### Dry deposition to the wetland

The two-layer model^38^ was used to determine dry deposition onto the wetland. Here a convenient reference height (usually 10 m) was divided into a constant (upper) layer and a deposition (lower) layer. The two layers can be distinguished by relative humidity, which decreases with height in the deposition layer but remains nearly unchanged in the constant layer. In this research, the dry deposition layer was determined as the space spanning the wetland surface to a height of 6 m, in accordance with our pre-investigation. The deposition velocity can be calculated as the dry deposition velocity in the wetland as follows:


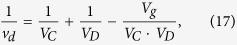


where *v*_*d*_ (cm s^−1^) is the deposition velocity, *V*_*g*_ can be calculated using Eq. (11), *V*_*C*_ (cm s^−1^) is the total transfer velocity in the constant flux layer, and *V*_*D*_ (cm s^−1^) is the total transfer velocity in the dry deposition layer. These parameters can be calculated as follows:


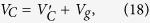



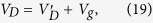


where 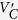 (cm s^−1^) and 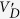 (cm s^−1^) are the transfer velocities without gravity in the constant flux layer and the dry deposition layer, respectively, and calculated as follows:


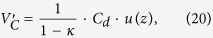



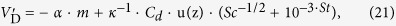


where *α* is a constant (10^3^ cm s^−1^/(g cm^−2^ s^−1^)), *m* is the rate of water evaporation, *C*_*d*_ is the drag coefficient, which is usually 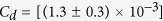 for wetlands with steady wind and near neutral stability, and Sc is the Schmidt number; more details on Sc can be found in Liu *et al*.^29^.

#### Statistical analysis

Data were analyzed using one-way analysis of variance (ANOVA) to test differences in FP accumulation among species; here, calculations for aquatic plants and trees were done separately. The accumulation capabilities of different species in both the surface and wax layers of leaves were also determined. One-way ANOVA was used to test differences in vegetation collection attributed to heights and species, as well as dry deposition in different seasons. Differences between individual means were tested using Tukey’s test, and the t-test was used to compare differences in the accumulation capacities of the two types of plants. The data met the assumptions of homogeneity of variances. All analyses were conducted using SPSS 22.0 (SPSS, Chicago, IL, USA).

## Results

### Leaf accumulation

**Figure 3:**
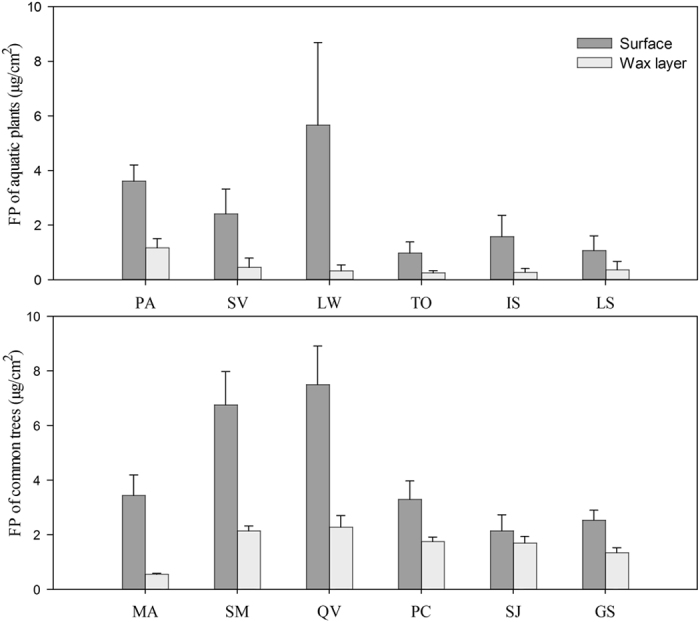
Accumulation of FP of plants: PA: *P. australis*, SV: *S. validus*, LW: *I. wilsonii*, TO: *T. orientalis*, IS: *I. setosa*, LS: *L. salicaria*, MA: *M. alba*, SM: *S. matsudana*, QV: *Q. variabilis*, PC: *P. Canadensis*, SJ: *S. japonica*, GS: *G. sinensis*, SC: *S. chinensis*.

Figure 3 describes the FP accumulation of each species on the surfaces and wax layers of leaves. The mean FP amounts of trees are higher than those of aquatic plants, but differences observed are not significant (P = 0.061). FP accumulation on tree species ranged from 3.85 μg cm ^−2^ to 9.78 μg cm ^−2^, with an average value of 5.91 μg cm ^−2^, while that on aquatic plants ranged from 1.23 to 5.98 μg cm ^−2^, with an average value of 3.03 μg cm ^−2^. Among the aquatic plants, *I. wilsonii* presents the highest mean accumulation (5.98 ± 3.24 μg cm ^−2^), followed by *P. australis* (4.78 ± 0.93 μg cm ^−2^); by contrast, *T. orientalis, I. setosa*, and *L. salicaria* show considerably lower accumulation values (less than 1.90 μg cm ^−2^). Among tree species, *Q. variabilis* (9.79 ± 1.82 μg cm ^−2^) and *S. matsudana* (8.90 ± 1.39 μg cm ^−2)^ present higher FP accumulations; the accumulations of *M. alba, S. japonica*, and *G. sinensis* are relatively lower (less than 4.0 μg cm ^−2^). While differences in accumulation on leaf surfaces between aquatic plants and trees are not significant (P = 0.176), those on wax layers are significant (P = 0.003); specifically, accumulation on the waxy layer of trees is higher than that on the waxy layers of aquatic plants. FP amounts accumulated on the leaf surface are much higher than those observed on the wax layer (P = 0.003). While differences in accumulation between the leaf surface and waxy layer of aquatic plants are fairly obvious, those for tree species such as *S. japonica* are relatively low.

### Structure of the leaf surface

**Figure 4:**
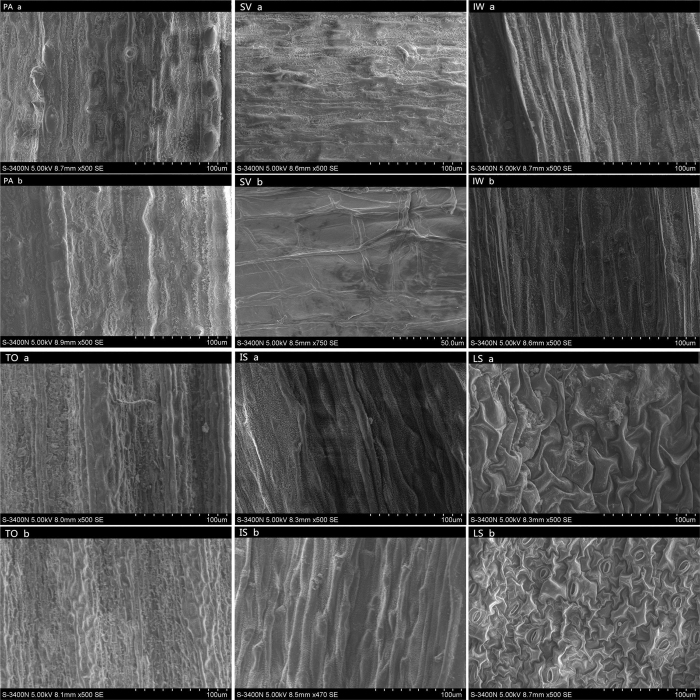
SEM photomicrographs of six aquatic plants (a: adaxial leave surface of species, b: abaxial leaf surface of species); most at ×500 SE, although abaxial leaf surface of *Scirpus validus* is ×750 SE and abaxial leaf surface of *Iris setosa* at ×470 SE).

**Figure 5:**
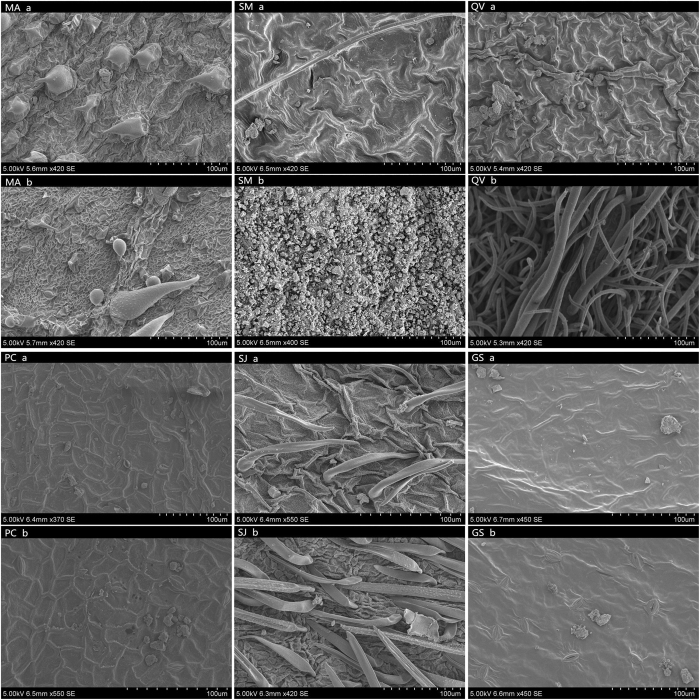
SEM photomicrographs of six common trees (a: adaxial leave surface of species, b: abaxial leaf surface of species; at ×370 SE – ×500 SE).

The structure of the leaf surface plays an important role in its ability to capture particles. Thus, different leaf structures may contribute to the heterogeneity of particle accumulation between different species. SEM photomicrographs of aquatic plants (Fig. 4) and common tree species (Fig. 5) show heterogeneity. Aquatic plants present simple grooves on their leaf surface, while tree leaves present more complicated structures, such as leaf hairs, protrusions, and raphe. Structural differences between species are also evident on their adaxial and abaxial surfaces (Figs 4 and 5).

### Vegetation collection and sedimentation

**Figure 6:**
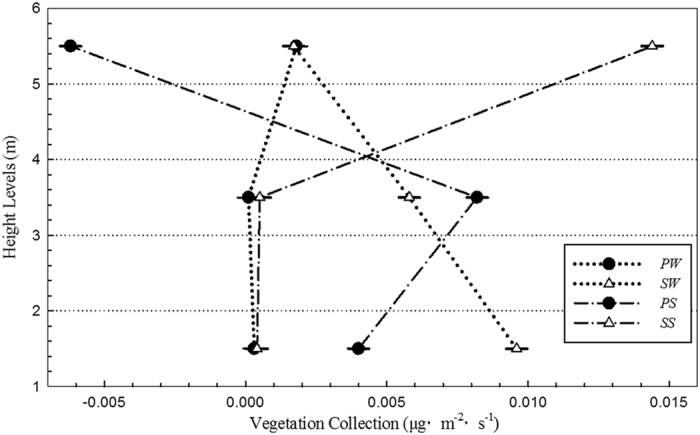
FP collections of two species at different height levels. SW: collection of *S. chinensis* in winter, PW: collection of *P. Canadensis* in winter, SS: collection of *S. chinensis* in summer, PS: collection of *P. Canadensis* in summer.

In the horizontal direction, FP movement was affected by meteorological conditions and the canopy properties. As FP moves from the windward direction to the leeward direction, it is collected by the canopy through impaction and interception. Figure 6 shows the amount of FP collected by *Populus* × *canadensis* and *S. chinensis* at different heights in the summer and winter. FP amounts collected at different heights show small differences (P = 0.801). Micro-climate conditions at different heights vary minimally, and canopy properties are heterogeneous for some species. Thus, no significant difference among the three heights investigated for the same species is observed.

In winter, the vegetation collection of *S. chinensis* (0.0057 ± 0.0040 μg m^−2^ s^−1^) is higher than that of *Populus* × *canadensis* (0.00073 ± 0.00093 μg m^−2^ s^−1^), and the difference observed is significant (P = 0.015); in summer, however, this difference is not significant (P = 0.408). Seasons promote no significant difference in the vegetation collection of *S. chinensis* (P = 0.913), but the collection amount of *Populus* × *canadensis* in summer is significantly higher than that in winter (P = 0.025). The maximum FP amounts collected by these two species occur in summer (*Populus* × *canadensis*, 0.0082 μg m^−2^ s^−1^; *S. chinensis*, 0.0144 μg m^−2^ s^−1^). *Populus* × *canadensis* is a deciduous species and its canopy is much denser in summer than in winter. The canopy of *S. chinensis* (an evergreen species) is also denser than that of *Populus* × *canadensis* in winter, and this difference may influence the variation in vegetation collection of these two species between seasons. In summer, differences among the two canopies do not appear to lead to significant differences in collection amount.

**Figure 7:**
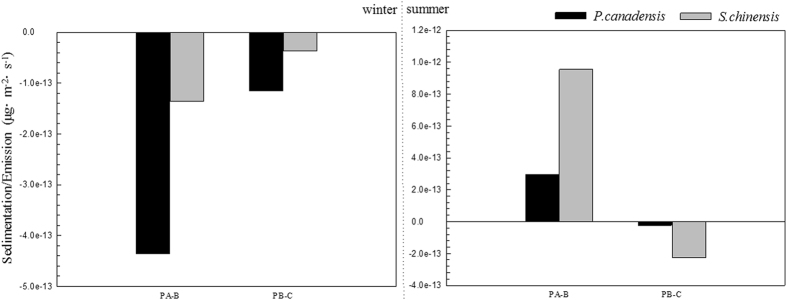
Sedimentation/emissions in two parts of the forest site at different height levels.

In the vertical direction, FP movements within the canopy are affected by both gravity and concentration gradient. FP is trapped within the canopy during hazy days or high FP concentration times. As the particle concentration beyond the canopy decreases, FP moves outward from the canopy. We used a model in the current study to obtain vector results and considered movement to be emission if its direction is from the canopy to the atmosphere; otherwise, movement was considered to be sedimentation. Figure 7 shows the sedimentation/emission of two species at different heights; here, calculated values are negative in winter but positive in summer. This result indicates that the forest is a source of FP in winter but a sink in summer. The absolute value of sedimentation/emission ((3.51 ± 3.98) × 10^−13^ μg m^−2^ s^−1^) is extremely small and much lower than the collection amount ((4.25 ± 4.50) × 10^−3^ μg m^−2^ s^−1^).

### Dry deposition

Table 1 summarizes FP dry deposition velocities in different seasons in the wetland and forest. P-values were calculated to indicate differences among various conditions (i.e., seasons, times, and land surfaces). In detail, the FP deposition velocity in the wetland is lower than that in the forest in summer, but the difference observed is not significant (P = 0.208). By contrast, in winter, the FP deposition velocity in the forest is significantly higher than that in the wetland (P = 0.018). Furthermore, no significant variation in deposition velocity between day and night is observed for both the wetland (P = 0.086) and forest (P = 0.610). Deposition velocities in winter (0.76 ± 0.31 cm s^−1^) are significantly higher than those in summer (0.42 ± 0.24 cm s^−1^, P = 0.001).

**Figure 8:**
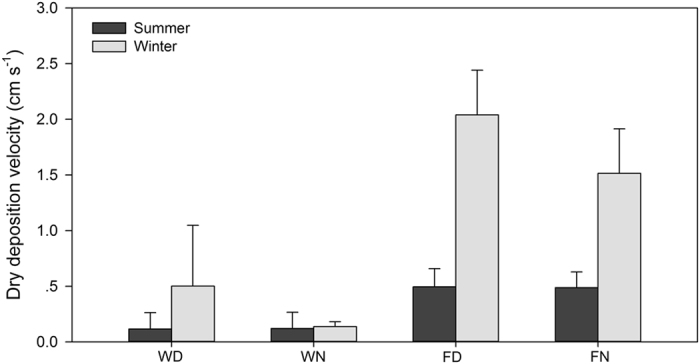
Dry deposition fluxes in different seasons and times in wetland and forest, where WD, WN, FD, and FN represent “daytime in wetland,” “nighttime in wetland,” “daytime in forest” and “nighttime in forest,” respectively.

Figure 8 shows the dry deposition fluxes of FP in the wetland and forest at different times. The respective deposition fluxes in the wetland and forest are 0.12 ± 0.15 μg m^−2^ s^−1^ and 0.49 ± 0.15 μg m^−2^ s^−1^ in summer and 0.32 ± 0.29 μg m^−2^ s^−1^ and 1.78 ± 0.40 μg m^−2^ s^−1^ in winter. In winter, deposition onto the forest is significantly higher than that onto the wetland (P = 0.012); dry deposition onto the forest is also significantly higher in this season than in summer (P = 0.004). Differences in deposition between seasons are not significant for the wetland (P = 0.104). In addition, variations between daytime and nighttime are not significant for either wetland (P = 0.684) or forest (P = 0.986).

## Discussion

### Leaf scale

Among the plants sampled, the tree species *S. matsudana* (8.90 ± 1.39 μg cm ^−2^) and *Q. variabilis* (9.79 ± 1.82 μg cm ^−2^) showed the highest accumulation of FP on leaf surfaces, while the aquatic species *T. orientalis* (1.23 ± 0.48 μg cm ^−2^) and *L. salicaria* (1.43 ± 0.85 μg cm ^−2^) showed the lowest accumulation of FM. In the current study, the mean FP accumulation of trees was significantly higher than that of aquatic species; however, the accumulation abilities of different species significantly differed (0.01 < P < 0.05), and the maximum accumulation was about 7.5 times that of the minimum. A previous study^6^ discussed the FP accumulation of 35 plant species (11 shrubs, 24 trees) in Beijing and showed no obvious difference between shrubs and trees in relation to accumulated particles; in this work, the average FP accumulation amount of trees was 4.51 μg cm ^−2^ while that of shrubs was 3.25 μg cm ^−2^. A study in Norway and Poland also compared the particle accumulation abilities of trees and shrubs and showed that the highest accumulation was 20 times more than the lowest^12^. While the uncertainty here must be highlighted, we assumed that 15 mm of precipitation could remove all of the particles pre-accumulated on the leaf surface in accordance with previous studies^11^,^32^ and our pre-experiments. This hypothesis requires further research under different weather and pollution backgrounds.

The complicated structures of tree leaves are considered to enable easier particle capture^39^; thus, tree species showed higher mean accumulation amounts than aquatic species. A leaf with larger numbers of stoma and leaf hairs can capture more particles than one with less of these structures^9^,^12^. *Populus* × *canadensis* presents a larger number of stoma on both of its leaf surfaces and is therefore able to capture more particles than *G. sinensis*, which presents virtually no stoma on its adaxial surface. *Q. variabilis* features high hair density on its abaxial surface and, therefore, higher FP accumulation than other species.

Certain studies have determined that the ability of the leaf surfaces of species such as *Kolkwitzia amabilis, Pyrus betulifolia*, and *Q. variabilis* to capture FP is related to their tomentose pubescence^6^. The hairy abaxial leaf surface of *Platanus occidentalis* has also been reported to be more efficient in accumulating particulate matter than a smooth adaxial leaf surface^40^. Different groove widths perform differently in capturing particles, and a previous study reported that the amount of particles collected increases with increasing groove width^9^. In contrast to these reports, however, in the present study, we found the opposite result: *T. orientalis* and *I. setosa* have wider grooves than *P. australis* and *I. wilsonii* but the former accumulates a smaller amount of FP than the latter. Surface roughness appeared to exert no significant effect on particle capture; *M. alba* and *S. matsudana*, for example, feature rough surfaces covered by a large number of protrusions on both sides, but these structures showed no significant correlation with the amount of FP accumulated by the leaves^12^.

This study only provides a qualitative analysis between leaf surface structures and particle accumulation; therefore, future studies may include quantification of the structures observed and determine the relationship between accumulation amounts and leaf structures. Similar to current and relevant studies, the aquatic species *P. australis* and *I. wilsonii* and the forest species *S. matsudana* and *Q. variabilis* performed very well in terms of accumulating particles. Considering these findings, we recommend that these species be the first selected for urban landscaping in areas of severe pollution.

### Canopy scale

Although the FP amount collected by vegetation is affected by the season, current research has determined these differences to be statistically insignificant. A study in an area of heavy traffic in Nanjing, China, showed the rank order of dust retained by trees in different seasons to be winter > autumn > summer > spring^41^. Another study in Qingdao, China, also reported that the dust-detaining capacity of ground cover plants presents a seasonal change in the order of winter > spring > autumn > summer^42^. In our study, the FP amount collected by *Populus* × *canadensis* in winter ((7.33 ± 7.60) × 10^−4^ μg m^−2^ s^−1^) was higher than that obtained in summer ((6.10 ± 2.10) × 10^−4^ μg m^−2^ s^−1^) but the difference in amounts was insignificant (P > 0.01). *S. chinensis* followed a similar pattern, showing collection amounts of (5.70 ± 3.23) × 10^−3^ μg m^−2^ s^−1^ in winter and (5.10 ± 6.58) × 10^−3^ μg m^−2^ s^−1^ in summer (P > 0.01).

Sedimentation in both *Populus* × *canadensis* and *S. chinensis* tree belts was negative in winter and positive in summer. The absolute values of emission in the evergreen conifer forest during winter were smaller than those in the deciduous broadleaf forest. Sedimentation is affected by gravity and accessible surfaces in the vertical direction^19^, which means it is also affected by leaf or foliage amounts. Few studies have focused on the quantitative relationship between vegetation collection and leaf/foliage amounts. In our study, the sedimentation amount was 10 orders of magnitude less than the vegetation amount collected; thus, sedimentation within the canopy can be ignored.

### Landscape scale

Wet and dry depositions on the landscape scale are considered to be two major pollutant removal processes^37^. Because of the relatively dry climate conditions (average annual precipitation, about 500 mm) in Beijing^43^, only dry deposition was estimated in this research. Forests and wetlands present unique biophysical properties that make their micro-climates and inter-surface conditions distinct, thereby leading to contrasting dry deposition results.

The dry deposition velocities in the forest and wetland calculated in the current study were compared with the results of previous studies in Beijing (Table 2). Although the values obtained were close, in this study, the dry deposition velocities in the forest revealed a large mean value and relatively small standard deviation, which means these velocities are fairly stable. Deposition velocities are known to depend strongly on atmospheric conditions. In contrast to previous studies that calculated deposition velocity over a relatively small time scale, in this study, a 12-hour average Monine-Obukhov length and friction velocity were used to achieve stable results; unfortunately, this technique also leads to an increase in uncertainty.

The results indicate that dry deposition velocities varied more dramatically between different periods within a single day while daily averages seemed to be more predictable. All of our results show that deposition velocities in the wetland are lower than those in the forest, which could be related to a number of micro-atmospheric conditions as well as the model structure applied. We acknowledge that our prediction model may not be 100% correct; thus, measurements must also be conducted. The results show that the deposition velocity at night is lower than that during the day, which is likely caused by relatively higher FP concentrations and friction velocities in the former than in the latter^29^,^31^. Deposition fluxes showed the opposite pattern, also likely because of the presence of higher FP concentrations in the day.

### Comparison of removal ability

On the leaf scale, the average FP accumulation of tree species was higher than that of aquatic species. The total leaf area of a forest is generally higher than that of a wetland of the same size; thus, forest plants are able to capture more FP than wetland plants. On the individual scale, coniferous species (*S. chinensis*) collected more FP than broadleaf species (*Populus* × *canadensis*), but the difference observed was statistically insignificant. On the landscape scale, significantly more FP was deposited onto the forest than onto the wetland, both in summer and winter, similar to the results reported by a previous study within the same area^7^,^29^. Forests are thus considered to remove more FP by dry deposition than other methods on the landscape scale.

Considering the results, forests in the urban area of Beijing present better FP removal ability than wetlands; thus, urban forests should be the first option in urban planning aiming to mitigate FP pollution. *S. matsudana* and *Q. variabilis* revealed better leaf accumulation ability than other trees, and coniferous species presented better removal ability (via vegetation collection and dry deposition) than aquatic species^31^. Despite these findings, wetlands remain indispensable landscapes in urban areas and provide a large number of other eco-service functions besides PM removal, such as flood storage, micro-climate regulation, and habitat provision^44^. In accordance with the results of this research, we suggest that *P. australis* and *I. wilsonii* be the preferred species used in wetland construction and that tree species be planted at perimeter areas, if possible. We note here that this work only focuses on FP collection. Deposition of other particles, such as PM10, on the surface of water in winter is known to be higher than that onto forests^7^,^29^. Consequently, further studies are required before a complete guide to urban planning for particle removal can be finalized.

### Uncertainty analysis

Dry deposition and vegetation collection velocities are highly influenced by particle size^4^,^19^,^33^,^45^. In the current study, we focused on particle sizes ranging from 0.2–2.5 μm; at this range, the deposition velocity varied minimally with particle size and the average diameter was used to calculate deposition and vegetation collection. Particle dry deposition is a dynamic process controlled by aerodynamic and meteorological conditions^9^,^46^, which may also be affected by air turbulence and other surface properties^13^. As such, using a simplified empirical parameter in the deposition and vegetation collection models could present another source of uncertainty. Despite this limitation, the calculation method used in this work has been proven to be valid because all of the errors and results obtained are in the same order of magnitude as those presented in related studies in the same area^7^,^29^,^30^,^31^. Thus, these models can adequately represent overall deposition.

## Conclusions

The FP removal ability of forests is better than that of wetland areas in the urban region of Beijing. On the leaf scale, common afforestation species are able to accumulate more FP on the leaf surfaces than aquatic species in wetlands. Among the species sampled in the urban forest and wetland ecosystem, *Q. variabilis* and *I. wilsonii* are the most effective in accumulating FP in their leaves. Horizontal vegetation collection is the major FP removal process on the individual scale; the contribution of vertical sedimentation/emission on this scale may be ignored. Coniferous tree species present stronger FP collection ability than broadleaf species, although differences between species are statistically insignificant. On the landscape scale, greater deposition onto the forest than onto the wetland may be observed. Furthermore, dry deposition is the major process of FP removal on rain-free days. In conclusion, when planning an urban green system, planting an urban forest should be the first option for FP mitigation.

## Additional Information

**How to cite this article:** Zhang, Z. *et al*. Multi-scale comparison of the fine particle removal capacity of urban forests and wetlands. *Sci. Rep.* **7**, 46214; doi: 10.1038/srep46214 (2017).

**Publisher's note:** Springer Nature remains neutral with regard to jurisdictional claims in published maps and institutional affiliations.

## Figures and Tables

**Table 1:** FP dry deposition velocities (cm s^−1^) at different times above wetland and forest.

	Summer	Winter
WN	0.05 ± 0.03	0.09 ± 0.07
WD	0.09 ± 0.05	0.16 ± 0.06
FN	0.60 ± 0.48	1.43 ± 0.28
FD	0.94 ± 0.42	1.36 ± 0.84

All values indicate “mean ± standard deviation,” and WD, WN, FD, FN stand for “daytime in wetland,” “nighttime in wetland,” “daytime in forest,” and “nighttime in forest”.

**Table 2:** Comparison between FP dry deposition velocities (cm s^−1^) in different seasons and at different times.

		Summer		Winter	
		Daytime	Nighttime	Daytime	Nighttime
Forest	Current Study	0.94 ± 0.42	0.60 ± 0.48	1.36 ± 0.84	1.43 ± 0.28
	Liu *et al*. 2016	0.98 ± 2.11	—	1.35 ± 2.14	—
	Sun *et al*. 2014	0.9 ± 0.8	0.4 ± 0.5	1.2 ± 1.7	0.7 ± 0.7
Wetland (Lake)	Current Study	0.09 ± 0.05	0.05 ± 0.03	0.16 ± 0.06	0.09 ± 0.07
	Liu *et al*. 2016	0.08 ± 0.06	—	0.50 ± 0.35	—
	Qiu *et al*. 2015	0.04 ± 0.05	0.10 ± 0.09	0.15 ± 0.11	0.17 ± 0.12
